# Design and Experimental Evaluation of a Peptide Antagonist against Amyloid β(1–42) Interactions with Calmodulin and Calbindin-D28k

**DOI:** 10.3390/ijms23042289

**Published:** 2022-02-18

**Authors:** Jairo Salazar, Joana Poejo, Ana M. Mata, Alejandro K. Samhan-Arias, Carlos Gutierrez-Merino

**Affiliations:** 1 Instituto de Biomarcadores de Patologías Moleculares, Universidad de Extremadura, 06006 Badajoz, Spain; jairochemsalazar@gmail.com (J.S.); joanapoejo86@gmail.com (J.P.); anam@unex.es (A.M.M.); 2 Departamento de Química, Universidad Nacional Autónoma de Nicaragua-León, León 21000, Nicaragua; 3 Departamento de Bioquímica y Biología Molecular y Genética, Facultad de Ciencias, Universidad de Extremadura, 06006 Badajoz, Spain; 4 Department of Biochemistry, Faculty of Medicine, Universidad Autónoma de Madrid (UAM), Arzobispo Morcillo, 4, 28029 Madrid, Spain; alejandro.samhan@uam.es; 5 Instituto de Investigaciones Biomédicas ‘Alberto Sols’ (CSIC-UAM), Arturo Duperier, 4, 28029 Madrid, Spain

**Keywords:** Amyloid β, calmodulin, calbindin-D28k, antagonist peptide, Alzheimer’s disease, fluorescence, docking

## Abstract

Amyloid β_1–42_ (Aβ(1–42)) oligomers have been linked to the pathogenesis of Alzheimer’s disease (AD). Intracellular calcium (Ca^2+^) homeostasis dysregulation with subsequent alterations of neuronal excitability has been proposed to mediate Aβ neurotoxicity in AD. The Ca^2+^ binding proteins calmodulin (CaM) and calbindin-D28k, whose expression levels are lowered in human AD brains, have relevant roles in neuronal survival and activity. In previous works, we have shown that CaM has a high affinity for Aβ(1–42) oligomers and extensively binds internalized Aβ(1–42) in neurons. In this work, we have designed a hydrophobic peptide of 10 amino acid residues: VFAFAMAFML (amidated-C-terminus amino acid) mimicking the interacting domain of CaM with Aβ (1–42), using a combined strategy based on the experimental results obtained for Aβ(1–42) binding to CaM and in silico docking analysis. The increase in the fluorescence intensity of Aβ(1–42) HiLyte^TM^-Fluor555 has been used to monitor the kinetics of complex formation with CaM and with calbindin-D28k. The complexation between nanomolar concentrations of Aβ(1–42) and calbindin-D28k is also a novel finding reported in this work. We found that the synthetic peptide VFAFAMAFML (amidated-C-terminus amino acid) is a potent inhibitor of the formation of Aβ(1–42):CaM and of Aβ(1–42):calbindin-D28k complexes.

## 1. Introduction

Amyloid β_1–42_ (Aβ(1–42)) oligomers have been linked to Alzheimer’s disease (AD) pathogenesis and suggested to be the cause of neuronal damage [1,2,3]. Also, it has been shown that neuronal uptake and accumulation of Aβ(1–42) aggregates correlate with metabolic inhibition [4]. Indeed, intraneuronal amyloid β (Aβ) accumulation precedes the appearance of amyloid plaques or tangles in transgenic mice models of AD [5,6,7,8]. In addition, the microinjection of Aβ(1–42) or cDNA encoding Aβ(1–42) has been shown to be neurotoxic to cultures of human neurons [9]. The dysregulation of intracellular calcium (Ca^2+^) homeostasis with subsequent alterations in neuronal excitability has been proposed to mediate Aβ neurotoxicity in AD [10,11,12]. Moreover, many studies have shown that Aβ(1–42) oligomers modulate the activity of systems that play a major role in the control of neuronal intracellular Ca^2+^ homeostasis, reviewed in [13].

The Ca^2+^-binding protein calmodulin (CaM) is present at high concentrations in neurons and reaches micromolar concentrations in cortical and hippocampal neurons, which have a high expression of this protein [14,15]. These neurons are in the brain areas that are highly prone to neurodegeneration in AD. In a previous work, we have shown that CaM binds to Aβ(1–42) oligomers and Aβ(25–35) peptide, with a dissociation constant of the Aβ:(Ca^2+^)_4_-CaM complex close to 1 nM [16]. Therefore, CaM has a high capacity to buffer the intracellular concentrations of these highly neurotoxic Aβ peptides. More recently, we have shown that CaM binds most of the Aβ(1–42) internalized in cerebellar granule neurons in culture after 2 h incubation with 2 μM of Aβ(1–42) oligomers added to the extracellular medium [17]. Dissociation constants of approximately 1 nM have been reported only for the complexes between Aβ peptides with two other proteins expressed in brain neurons, namely, cellular prion protein [18] and glycogen synthase kinase 3α [19]. Since the concentration of CaM in neurons is orders of magnitude higher than that of these proteins, we can conclude that CaM can act as a major neuronal sink for neurotoxic intracellular Aβ peptides. As CaM has a major regulatory role in neuronal metabolism, excitability, and intracellular calcium signaling [12,13,20], the possibility that Aβ(1–42):CaM complexes could also function as intracellular transducers for the focalized actions of Aβ peptides emerges from our previous works.

Calbindin-D28k is also a major Ca^2+^ binding protein that is highly expressed in the brain [21] and plays a major role in the control of the resting cytosolic Ca^2+^ concentration in hippocampal neurons. In fact, the inhibition of calbindin-D28k expression elicits a prolonged increase in intraneuronal Ca^2+^ concentration after N-methyl-D-aspartate or potassium stimulation of hippocampal slices [22]. It should be noted that calbindin-D28k protein levels are markedly reduced in brain samples from human AD and rodent models of AD [23]. The calbindin-D28k-expressing cells in the brains of AD patients have been shown to be more resistant to degeneration [24]. In glial and neuronal cells, the overexpression of calbindin-D28k has been shown to inhibit the apoptosis induced by Aβ and the mutant presenilin-1 detected in AD [25,26]. In addition, the calbindin-D28k knock-out of transgenic mice with 5 familial AD mutations (5XFAD;Tg) aggravates AD pathogenesis, suggesting that calbindin-D28k has a critical role in AD pathogenesis [27].

The use of CaM knock-out animal models or CaM si-RNA in cultured neurons is strongly hampered by the high relevance of these proteins to brain development and normal neuronal function, respectively. This severely limits our knowledge of the relative contribution of the many different putative molecular mechanisms underlying intracellular Ca^2+^ dysregulation by internalized Aβ(1–42). Since there is a wide range of intracellular signaling pathways modulated by CaM in neurons, there is a need for alternative strategies to experimentally evaluate the effects of Aβ(1–42):CaM complexation in neuronal activity and also in neuronal plasticity and survival. On the other hand, CaM is a small and water-soluble protein whose high-resolution, three-dimensional structure is available in the UniProt Protein Data Bank, as well as that of Aβ(1–42) monomer. This fact allows for the performance of docking simulations between them, opening the possibility of designing high affinity antagonist polypeptides against this interaction. Indeed, it is known that several endogenous neuropeptides can antagonize the actions of Aβ, both in animal models [28,29,30] and in cell cultures [31,32]. Furthermore, in our previous study of Aβ(1–42):CaM complexation, we concluded that the Aβ(25–35) peptide interaction with CaM is largely dominated by hydrophobic interactions between the uncharged amino acids of both the peptide and CaM [16]. Due to this, it is likely that the amino acid sequence of Aβ(1–42) that is directly involved in the formation of the Aβ(1–42):CaM complex could also be implicated in the complexation of Aβ(1–42) with other neuronal proteins, although with lower affinity. While Aβ(1–42):CaM complex formation may account for, at least in part, the large range of subcellular signaling pathways reported to be altered by intracellular and extracellular Aβ(1–42), this is a point that still deserves to be experimentally assessed.

On these grounds, the main goal of this work has been to design a peptide that can antagonize the formation of the Aβ(1–42):CaM complex and experimentally evaluate its efficiency to prevent the complexation of Aβ(1–42) with CaM and with calbindin-D28k. To this end, we have set up a fluorescence assay to monitor the formation of the complex of Aβ(1–42) with purified recombinant human CaM. Using this approach, we show that Aβ(1–42) also binds to calbindin-D28k, although with a slightly lower affinity than to CaM. Then, we have used a docking strategy to simulate the most probable model structures of the Aβ(1–42):CaM complex that meet the experimental requirements derived from our previous work [16] and to design synthetic peptides that can antagonize the formation of complexes between Aβ(1–42):CaM and Aβ(1–42):calbindin-D28k. 

## 2. Results

### 2.1. Interaction between Aβ(1–42) HiLyte^TM^-Fluor555 and Calmodulin (CaM)

**Figure 1:**
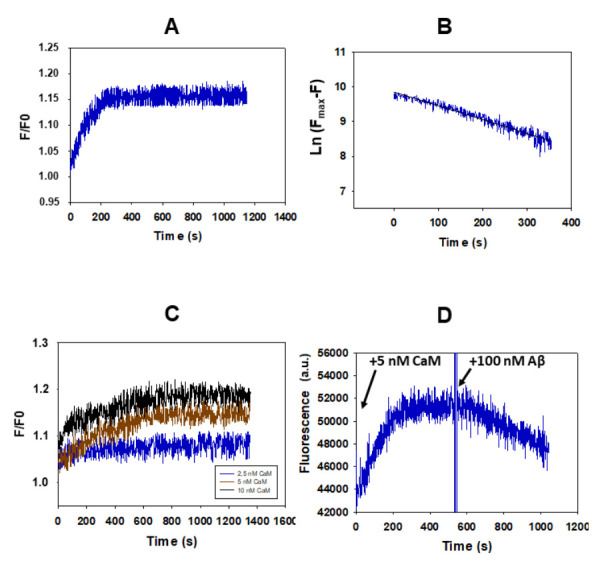
The kinetics of complex formation between Aβ(1–42) and CaM monitored by the increase in the fluorescence of Aβ(1–42) HiLyte^TM^-Fluor555. (**A**) A representative kinetic recording of the increase in the fluorescence intensity of 10 nM of Aβ(1–42) HiLyte^TM^-Fluor555 after the addition of 5 nM of CaM. (**B**) The increase in fluorescence of Aβ(1–42) HiLyte^TM^-Fluor555 has been fit to a first-order kinetic process. F_max_ is the maximum fluorescence intensity at the completion of the kinetic process. The black continuous line is the linear least-squares fit of the data of the panel A to the equation: y = 9.845 − 0.00396 x (R^2^ = 0.922). (**C**) The dependence of the kinetics of increase in fluorescence intensity of 10 nM of Aβ(1–42) HiLyte^TM^-Fluor555 upon the following concentrations of CaM: 2.5 nM (blue), 5 nM (brown) and 10 nM (black). (**D**) A reversion of the increase in fluorescence of Aβ(1–42) HiLyte^TM^-Fluor555 elicited by 5 nM of CaM by the addition of 100 nM of Aβ(1–42) at the point indicated by an arrow. The fluorescence measurements have been performed as indicated in the Materials and Methods, and a.u. means fluorescence units given by the fluorimeter readings.

In a previous work [16] we found that titration of the fluorescent Aβ(1–42) HiLyte^TM^-Fluor555 with nanomolar CaM concentrations enhances the fluorescence intensity. The results of the Figure 1A show that the addition of 5 nM of CaM to a 10 nM of Aβ(1–42) HiLyte^TM^-Fluor555 solution elicits a kinetics of increase in the fluorescence intensity of the HiLyte^TM^-Fluor555 dye of 20–25%, in good agreement with the results reported in [16]. This allows for the monitoring of the kinetics of interaction between both molecules and points out that the microenvironment of the dye is significantly altered upon formation of the Aβ(1–42) HiLyte^TM^-Fluor555:CaM complex. Also, it is to be noted that this complex formation does not produce a significant shift in the emission spectra of Aβ(1–42) HiLyte^TM^-Fluor555 (data not shown). The increase in fluorescence as a function of time after the addition of CaM follows first-order kinetics (Figure 1B), with a rate constant (k_on_) of (4.0 ± 0.3)·10^−3^ s^−1^, i.e., with a half-time of 173 ± 10 s.

Next, we studied this kinetic process with 10 nM of Aβ(1–42) HiLyte^TM^-Fluor555 at different concentrations of CaM, i.e., by varying the molar ratio of Aβ(1–42):CaM (Figure 1C). These results revealed that the maximum fluorescence change was achieved when CaM concentration increased from 2.5 to 5 nM, and it is not significantly different between 5 and 10 nM of CaM. It is noteworthy that at 5 nM of CaM the molar ratio is 2:1, and in a previous work, we concluded that CaM binds Aβ(1–42) dimers [16]. Thus, the results shown in the Figure 1C lend further support to this hypothesis, because Aβ(1–42) is largely in a dimeric state in our experimental conditions, as indicated in the Materials and Methods section. However, with a 1:1 molar ratio of Aβ(1–42):CaM, i.e., 10 nM of CaM and 10 nM of Aβ(1–42), the rate constant of the kinetic process increases about 4-fold, up to (18 ± 4)·10^−3^ s^−1^ (half-time 38 ± 7 s), suggesting that a second CaM molecule can also bind to the Aβ(1–42)_2_:CaM complex, leading to Aβ(1–42)_2_:CaM_2_ complexes. 

The previous kinetic studies allowed us to obtain the rate of formation of the Aβ(1–42) HiLyte^TM^-Fluor555:CaM complex, and also pointed out a high affinity of CaM for Aβ(1–42), resulting in extensive complexation in the nanomolar concentration range of both partners. In order to evaluate the dissociation constant of the Aβ(1–42) HiLyte^TM^-Fluor555:CaM complex, we have measured the kinetics of reversion of the increase in fluorescence upon addition of 100 of nM of non-labelled Aβ(1–42) after the completion of the kinetics of Aβ(1–42) HiLyte^TM^-Fluor555:CaM complex formation under the same experimental conditions (Figure 1D). The decrease in the fluorescence monitors the kinetics of exchange of Aβ(1–42) HiLyte^TM^-Fluor555 by non-labelled Aβ(1–42). It can be seen that this is a much slower kinetic process, since the time needed for the decay to half the increase of fluorescence is 900 ± 50 s, and we have calculated a rate constant of the exchange of Aβ(1–42) HiLyte^TM^-Fluor555 by non-labelled Aβ(1–42) (k_off_) of (7.7 ± 0.9)·10^−4^ s^−1^. Thus, our results led to a value of about 5 for the ratio between the average rate constant of the rate-limiting steps of complex formation and dissociation (k_on_/k_off_). This pointed out that at nanomolar concentrations of Aβ(1–42) and CaM, the equilibrium is largely displaced towards the Aβ(1–42):CaM complex formation.

### 2.2. Docking between Aβ(1–42) and CaM and the Criteria to Select the Most Probable in Silico Structural Models of the Aβ(1–42):CaM Complex

**Figure 2:**
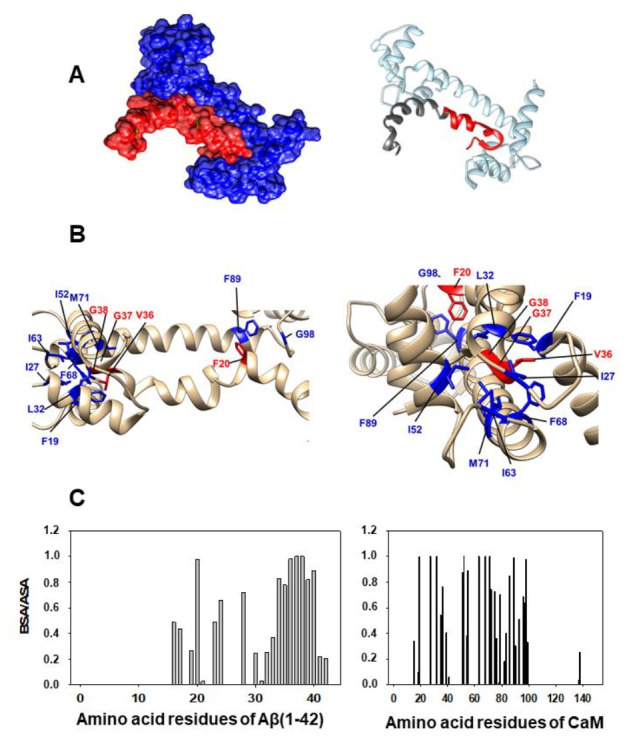
The selected in silico model structure 1 of the complex Aβ(1–42):CaM obtained using the PDB ID files: 1Z0Q for Aβ(1–42) and 1CLL for Ca^2+^-saturated CaM. Docking was performed as indicated in the Materials and Methods section. (**A**) The space-filled and peptide and protein backbone images of the complex. Aβ(1–42) is stained in red and CaM is stained in blue in the space-filled image. The protein backbone of CaM is stained in light blue, the 24–42 amino acid residues of Aβ(1–42) are stained in red, and the rest of the Aβ(1–42) is stained in dark grey. (**B**) The structural details of the Aβ(1–42):CaM interface of in silico model structure 1: a side view on the left and a front view on the right. The CaM amino acid residues (colored in blue) more strongly interact with the 24–42 amino acid residues of Aβ(1–42) (colored in red). (**C**) Graphic charts of the BSA/ASA values obtained for the amino acids residues of Aβ(1–42) and CaM in the in silico model structure 1 of the Aβ(1–42):CaM complex.

**Figure 3:**
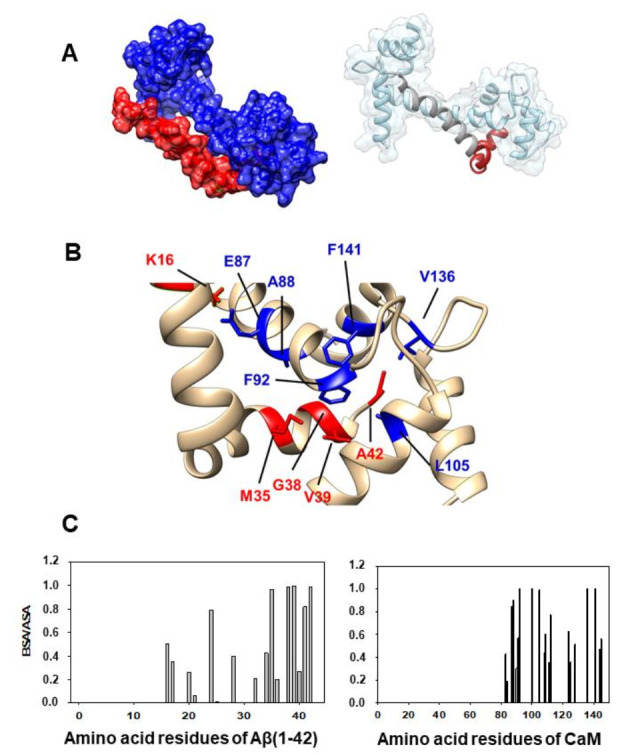
The selected in silico model structure 2 of the complex Aβ(1–42):CaM obtained using the PDB ID files: 1IYT for Aβ(1–42) and 1CLL for Ca^2+^-saturated CaM. Docking was performed as indicated in the Materials and Methods section. (**A**) The space-filled and peptide and protein backbone images of the complex. Aβ(1–42) is stained in red and CaM is stained in blue in the space-filled image. The protein backbone of CaM is stained in light blue, the 24–42 amino acid residues of Aβ(1–42) are stained in red, and the rest of the Aβ(1–42) is stained in dark grey. (**B**) The structural details of the Aβ(1–42):CaM interface of in silico model structure 2. The CaM amino acid residues (colored in blue) more strongly interact with the 24–42 amino acid residues of Aβ(1–42) (colored in red). (**C**) Graphic charts of the BSA/ASA values obtained for the amino acids residues of Aβ(1–42) and CaM in the in silico model structure 2 of the Aβ(1–42):CaM complex.

Docking between Aβ(1–42) and CaM has been performed following the methodology indicated in the Materials and Methods section. First, we obtained the PDB files of the 10 most probable structures after Gibbs free energy minimization (ΔG) given by the ClusPro server for the Aβ(1–42) PDB ID: 1IYT and 1Z0Q files. Taking into account the experimental results obtained in our previous work on Aβ(1–42):CaM complex formation [16], we considered acceptable only the simulations for the Aβ(1–42):CaM complex that presented strong hydrophobic interactions between the amino acids residues of the peptide in the 25–35 segment. Also, our previous work showed that Aβ(1–42):CaM complexation does not alter Ca^2+^ binding to CaM [16], excluding that Aβ(1–42) binds to the Ca^2+^-binding domains of CaM. The application of these criteria reduced the number of most probable model structures for the complex formation between Aβ(1–42) and CaM to 4. Then, we selected two highly ranked hydrophobic-favored models, one for each Aβ(1–42) PDB ID file, applying the criteria of the lowest ΔG values among the models that showed extensive overlap with the CaM domain interacting with the Aβ(25–35) peptide shown in Figure 4 of ref. [16]. The interacting interface of the two selected models was analyzed using PDBePISA (Protein Interfaces, Surfaces, and Assemblies). We named our models as the Structures 1 and 2 shown in the Figure 2 and Figure 3, respectively. The ΔG values obtained for structures 1 and 2 were −17 and −15 kcal/mol, which are consistent with the low dissociation constant of the Aβ(1–42):CaM complex reported in our previous experimental work [16]. The in silico analysis of the interacting interface is summarized in Table 1, which shows the most likely amino acid residues of Aβ(1–42) and CaM involved in the interactions established for both model structures of the Aβ(1–42):CaM complex. To be conservative, we have included in these lists only those amino acid residues of Aβ(1–42) and CaM with BSA/ASA ≥ 0.5, i.e., well above the threshold recommended value of 0.15. 

### 2.3. Design of an Antagonist Peptide for the Formation of the Aβ(1–42):CaM Complex and Its Experimental Evaluation

In order to design a peptide antagonist for the complex formation between Aβ(1–42) and CaM, we first identified the amino acid residues of CaM directly interacting with the amino acid residues of the 24–42 peptide domain of Aβ(1–42) in the previously selected docking simulations (structures 1 and 2), see the detail of the structures 1 and 2 shown in the Figure 2B and Figure 3B. The data shown in the Table 1 highlight the most relevant amino acids residues of Aβ(1–42) and CaM found in the analysis of the interface using the simulated model structures of the Aβ(1–42):CaM complex in silico. We choose the structure 1 of the Aβ(1–42) and CaM as the template structure for the design of the peptide, because the prediction shows more amino acid residues in the 24–42 domain of Aβ(1–42) strongly interacting with CaM, as pointed out by the average higher values of the parameter BSA/ASA obtained from the interface analysis with PDBePISA software (Table 1), and, also, have a more favorable ΔG value. Next, we calculated the separation distance between the CaM amino acid residues participating in these pairs in the three-dimensional CaM structure. Thereafter, using standard amino acid residues volume size, we introduced non-polar amino acid residues to yield a separation between amino acid residues mimicking their separation in the three-dimensional CaM structure. This operational protocol led us to the following ten amino acid residues peptide (from N-terminus to C-terminus): VFAFAMAFML (amidated-C-terminus amino acid) as a putative peptide antagonist for the formation of the Aβ(1–42):CaM complex.

**Figure 4:**
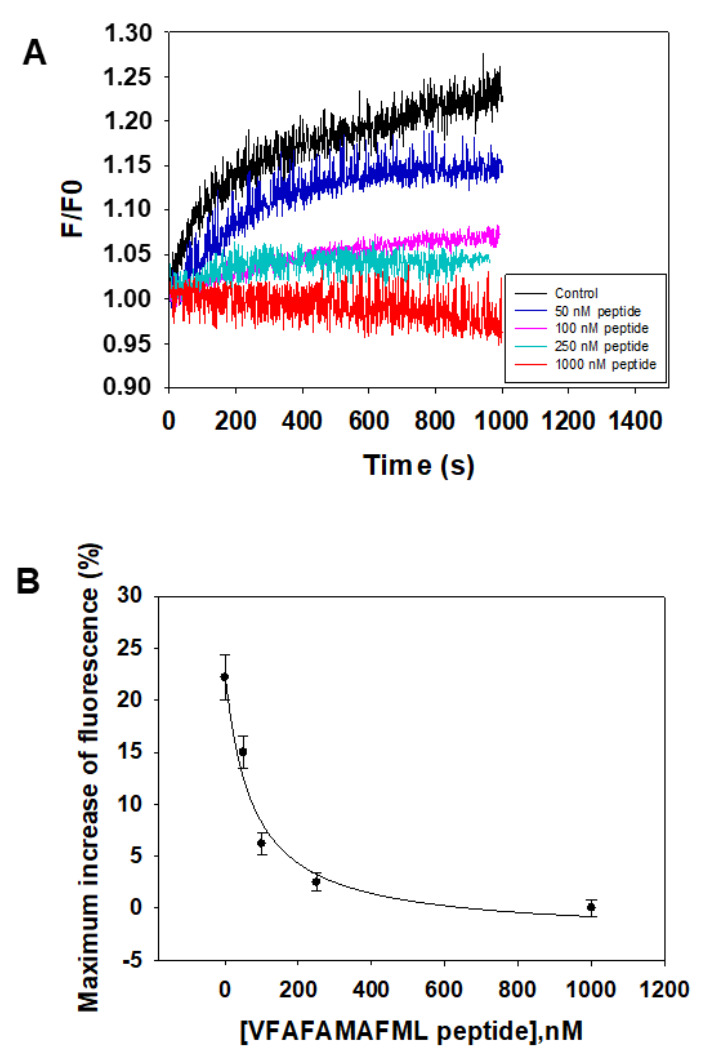
The synthetic peptide VFAFAMAFML (amidated-C-terminus amino acid) antagonizes Aβ(1–42):CaM complex formation. (**A**) Effect of increasing concentrations of this synthetic peptide on the kinetics of increase of the fluorescence intensity of 10 nM Aβ(1–42) HiLyte^TM^-Fluor555 after the addition of 5 nM CaM. Titration with the synthetic peptide and fluorescence measurements have been performed as indicated in the Materials and Methods. The kinetics of fluorescence were recorded in the presence of the following concentrations of the peptide VFAFAMAFML (amidated-C-terminus amino acid): 0 (black), 50 nM (dark blue), 100 nM (pink), 250 nM (cyan) and 1000 nM (red). (**B**) Plot of the dependence of the maximum increase of fluorescence recorded 1000 s after the addition of CaM as a function of the concentrations of the peptide VFAFAMAFML (amidated-C-terminus amino acid). The data shown are the average (black-filled circles) ± S.E. (error bars) of the results obtained in triplicate measurements. The continuous line is the non-linear square fit of the data to the hyperbolic equation for a one site binding drug-inhibitory effect: Y = Y0 − [Qmax × x/(IC50 + x)], where Y0 is the maximum fluorescence increase in the absence of the peptide, Qmax is the maximum quenching of the increase of fluorescence at saturation of the peptide and IC50 is the concentration of the peptide that reduces to half the maximum increase of fluorescence. The data fitting gave the following results: R^2^ = 0.966, Y0 = 22%, Qmax = 22% and IC50 = 75 ± 10 nM.

The synthetic peptide VFAFAMAFML (amidated-C-terminus amino acid) has been tested for its ability to antagonize Aβ(1–42):CaM complex formation using Aβ(1–42) HiLyte^TM^-Fluor555. The results (Figure 4) show that only submicromolar concentrations of this peptide are needed to inhibit the kinetic of fluorescence increase that monitors the interaction between 10 nM Aβ(1–42) HiLyte^TM^-Fluor555 and 5 nM CaM. Indeed, 1 μM of the peptide VFAFAMAFML (amidated-C-terminus amino acid) completely blocks the kinetics of fluorescence increase. An IC50 value of 75 ± 10 nM can be calculated for this peptide from these results, as indicated in the legend of the Figure 4B. 

### 2.4. Interaction between Aβ(1–42) HiLyte^TM^-Fluor555 and Calbindin-D28k

**Figure 5:**
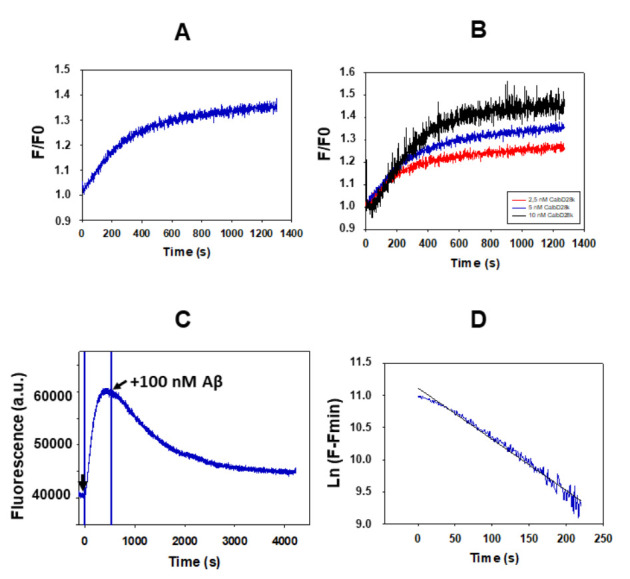
Kinetics of complex formation between Aβ(1–42) and calbindin-D28k monitored by the increase of the fluorescence of Aβ(1–42) HiLyte^TM^-Fluor555. (**A**) Representative kinetic recording of the increase of the fluorescence intensity of 10 nM Aβ(1–42) HiLyte^TM^-Fluor555 after the addition of 5 nM calbindin-D28k. (**B**) Dependence of the kinetics of increase of fluorescence intensity of 10 nM Aβ(1–42) HiLyte^TM^-Fluor555 upon the concentration of calbindin-D20k: 2.5 nM (red), 5 nM (blue) and 10 nM (black). (**C**) The increase of fluorescence of Aβ(1–42) HiLyte^TM^-Fluor555 after addition of 5 nM calbindin-D28k (first arrow) is reversed by the addition of 100 nM Aβ(1–42) at the point indicated by the second arrow. (**D**) The decay of the fluorescence of Aβ(1–42) HiLyte^TM^-Fluor555 after the addition of 100 nM Aβ(1–42) follows a first-order kinetic process with an initial lag phase of around 60 s. Fmin is the minimum fluorescence intensity at the completion of the kinetic process. The black continuous line is the linear least-squares fit of the data of the panel C to the equation: y = 11.12 − 0.007998x (R^2^ = 0.982). Fluorescence measurements have been performed as indicated in the Materials and Methods, and a.u. means fluorescence units given by the fluorimeter readings.

The increase of fluorescence intensity of the HiLyte^TM^-Fluor555 dye after the addition of 5 nM calbindin-D28k to a 10 nM Aβ(1–42) HiLyte^TM^-Fluor555 solution is shown in the Figure 5A. Our results show that the change in fluorescent intensity is even higher than that found with CaM (see Figure 1A). Thus, the results of the fluorescence kinetics which monitors the interaction between calbindin-D28k point out that the microenvironment of the dye is more significantly altered upon formation of the complex of Aβ(1–42) HiLyte^TM^-Fluor555 with calbindin-D28k than with CaM. Also, it should be highlighted that this complex formation do not produce a significant shift of the wavelength of the major emission band of the spectra of Aβ(1–42) HiLyte^TM^-Fluor555 (data not shown). Noteworthy, the kinetics of increase of fluorescence observed after addition of calbindin-D28k to a 10 nM Aβ(1–42) HiLyte^TM^-Fluor555 solution has an initial lag phase of about 1 min, which was not observed in the kinetics after the addition of CaM. This result suggests an initial conformational shift in the Aβ(1–42):calbindin-D28k that ensues a tighter interaction between both molecules. Consistent with this, the time needed to reach 50% of the maximum increase of the fluorescence of Aβ(1–42) HiLyte^TM^-Fluor555 is higher than that found with CaM, namely, 250 ± 10 s for calbindin-D28k versus 173 ± 10 s for CaM (Figure 1A and Figure 5A). 

Figure 5B shows the dependence of this kinetic process upon the addition of different concentrations of calbindin-D28k. These results revealed that the maximum change of fluorescence increases when calbindin-D28k concentration increases from 2.5 up to 10 nM. At 10 nM calbindin-D28k concentration the molar ratio Aβ(1–42): calbindin-D28k is 1:1. Since Aβ(1–42) is largely in dimeric state in our experimental conditions, as indicated in the Materials and Methods section, these results lend support to the hypothesis that complexes of (Aβ(1–42))_2_: (calbindin-D28k)_2_ are being formed.

The previous kinetics indicated a high affinity of calbindin-D28k for Aβ(1–42) resulting in extensive complexation in the nanomolar concentration range of both partners. This was confirmed by the reversion of the increase of fluorescence elicited by 5 nM calbindin-D28k upon addition of 100 nM of non-labelled Aβ(1–42) after completion of the kinetics of Aβ(1–42) HiLyte^TM^-Fluor555: calbindin-D28k complex formation (Figure 5C). The decrease of the fluorescence monitors the kinetics of exchange of Aβ(1–42) HiLyte^TM^-Fluor555 by non-labelled Aβ(1–42), and from these results the rate of dissociation of Aβ(1–42) HiLyte^TM^-Fluor555:calbindin-D28k complexes can be calculated. It can be seen that this is a slower kinetic process with a short lag time of 1–2 min, pointing out that a conformational relaxation of the complex is needed before the release of Aβ(1–42) HiLyte^TM^-Fluor555. The fit to a first-order kinetic process yields a maximum rate of the fluorescence decay of (8.3 ± 0.4)·10^−3^ s^−1^ (Figure 5D). Remarkably, this rate constant is about 10-fold higher than the rate constant obtained for this reversion kinetic process with CaM. In addition, the calculated ratio between the half-times of complex formation and dissociation is ≈3 for the Aβ(1–42):calbindin-D28k complexes, while the value obtained for Aβ(1–42):CaM is 0.2. Thus, our results allow us to conclude that CaM displays approximately 10-fold higher affinity for Aβ(1–42) than calbindin-D28k.

**Figure 6:**
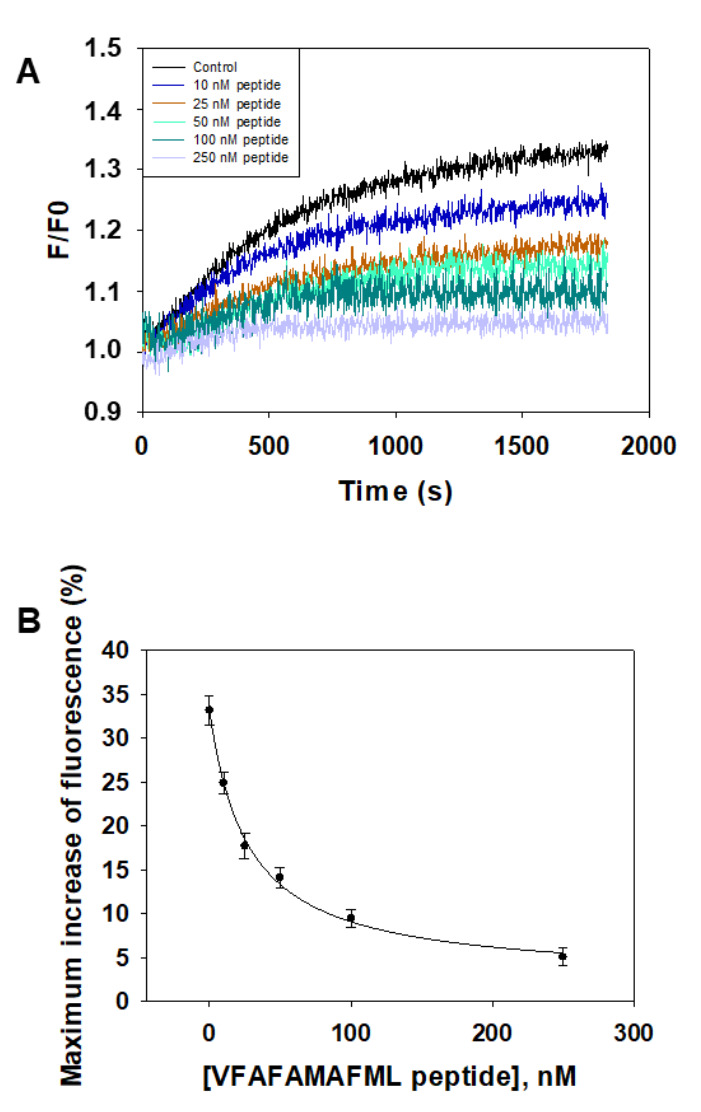
The synthetic peptide VFAFAMAFML (amidated-C-terminus amino acid) antagonizes Aβ(1–42):calbindin-D28k complex formation. (**A**) Effect of increasing concentrations of this synthetic peptide on the kinetics of increase of the fluorescence intensity of 10 nM Aβ(1–42) HiLyte^TM^-Fluor555 after the addition of 5 nM calbindin-D28k. Titration with the synthetic peptide and fluorescence measurements have been performed as indicated in the Materials and Methods. The kinetics of fluorescence were recorded in the presence of the following concentrations of the peptide VFAFAMAFML (amidated-C-terminus amino acid): 0 (black), 10 nM (dark blue), 25 nM (brown), 50 nM (cyan), 100 nM (green) and 250 nM (grey). (**B**) Plot of the dependence of the maximum increase of fluorescence recorded 1700 s after the addition of calbindin-D28k as a function of the concentrations of the peptide VFAFAMAFML (amidated-C-terminus amino acid). The data shown are the average (black-filled circles) ± S.E. (error bars) of the results obtained in triplicate measurements. The continuous line is the non-linear square fit of the data to the hyperbolic equation for a one site binding drug-inhibitory effect: Y = Y0 − [Qmax × x/(IC50 + x)], where Y0 is the maximum fluorescence increase in the absence of the peptide, Qmax is the maximum quenching of the increase of fluorescence at saturation of the peptide and IC50 is the concentration of the peptide that reduces to half the maximum increase of fluorescence. The data fitting gave the following results: R^2^ = 0.99694, Y0 = 33%, Qmax = 33% and IC50 = 27 ± 3 nM.

The synthetic peptide VFAFAMAFML (amidated-C-terminus amino acid) has been tested for its ability to antagonize Aβ(1–42):calbindin-D28k complex formation using Aβ(1–42) HiLyte^TM^-Fluor555 (Figure 6). The results show that only submicromolar concentrations of this peptide are needed to inhibit the kinetic of increase of fluorescence that monitors the interaction between 10 nM Aβ(1–42) HiLyte^TM^-Fluor555 and 5 nM CaM. Indeed, 250 nM of VFAFAMAFML (amidated-C-terminus amino acid) almost completely block the kinetics of increase of fluorescence. An IC50 value of 27 ± 3 nM can be calculated from these results, as indicated in the legend of the Figure 6B. 

### 2.5. Docking between Aβ(1–42) and Calbindin-D28k Gives Support to the Formation of Aβ(1–42):Calbindin-D28k Complexes

**Figure 7:**
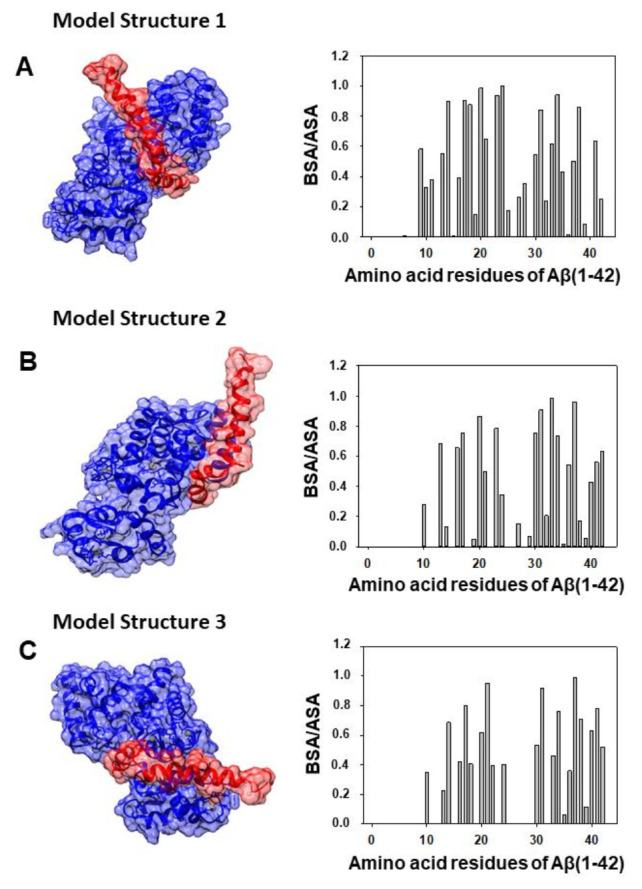
Selected in silico model structures of the complex Aβ(1–42):calbindin-D28k obtained using the PDB ID files: 1IYT for Aβ(1–42) and 6FIE for calbindin-D28k. (**A**) Model structure 1. (**B**) Model Structure 2. (**C**) Model structure 3. For each model structure of the Aβ(1–42):calbindin-D28k complex a space-filled image with highlighted peptide and protein backbones is shown. Aβ(1–42) is stained in red and calbindin-D28k is stained in blue in the space-filled image. The graphic charts of the BSA/ASA values obtained for the amino acids residues of Aβ(1–42) in the in silico model structures 1, 2 and 3 of the Aβ(1–42):calbindin-D28k complex are also shown in each panel (**A**–**C**) of this figure next (at the right-side) of each space-filled image. Docking was performed as indicated in the Materials and Methods.

As shown above, the hydrophobic peptide VFAFAMAFML (amidated-C-terminus amino acid) behaves as an antagonist against the formation of the Aβ(1–42):CaM complex and also against Aβ(1–42):calbindin-D28k complexes formation. This experimental observation led us to choose, within the highest-ranked hydrophobic-favored models yielded by the Cluspro server, only the simulations of the complex Aβ(1–42):calbindin-D28k displaying a large number of interactions with amino acid residues of the Aβ(1–42) selected for the design of this peptide antagonist. Docking between Aβ(1–42) and calbindin-D28k has been performed as indicated in the Materials and Methods section. As a result we found that docking with the Aβ(1–42) PDB ID: 1IYT structure gave us more simulations of the Aβ(1–42):calbindin-D28k complex than PDB ID: 1Z0Q with extensive interactions between the amino acid residues 24–42 of Aβ(1–42) and calbindin-D28k. This suggests that the microenvironment of the binding pocket of Aβ(1–42) in calbindin-D28k is higher than in CaM, which is also in good agreement with the higher increase of the fluorescence of Aβ(1–42) HiLyte^TM^-Fluor555 upon binding to calbindin-D28k. On these grounds, we obtained the PDB files of the 10 most probable structures after free energy minimization (ΔG) given by the ClusPro server for the Aβ(1–42) PDB ID: 1IYT, all with negative ΔG values higher than 10 kcal/mol. Therefore, docking simulations predict a high-affinity binding between Aβ(1–42) and calbindin-D28k, which is in good agreement with the results obtained in fluorescence kinetic experiments. Three of the most probable structures for the complex Aβ(1–42):calbindin-D28k showed extensive interactions between the 24–42 amino acid residues of Aβ(1–42) and calbindin-D28k. These are the model structures of the Aβ(1–42):calbindin-D28k complexes shown in the Figure 7, for which the docking simulation gives ΔG values of −17 (model 1), −12 (model 2) and −11.6 (model 3) kcal/mol. Then, we selected these structures with the lowest ΔG values for interface analysis with PDBePISA, which yielded the amino acid residues of Aβ(1–42) and calbindin-D28k listed in the Table 2 as those whose microenvironment is more strongly altered by the interactions established in the Aβ(1–42):calbindin-D28k complex in the three selected models.

## 3. Discussion

This work demonstrates that nanomolar concentrations of Aβ(1–42) efficiently interact with the calcium-saturated forms of CaM and calbindin-D28k leading to the formation of stable Aβ(1–42):CaM and Aβ(1–42):calbindin-D28k complexes. The latter result bears a particular relevance in brain neurons expressing calbindin-D28k. As noted in the introduction, this protein is abundant throughout the central nervous system including pyramidal hippocampal neurons and cortical neurons [33], which are in brain regions highly sensitive to neurodegeneration in AD. Since in a previous work [16] we found that the affinity of Aβ(1–42) for apo-CaM (Ca^2+^-free CaM) is about 20-fold lower than for CaM saturated by Ca^2+^, we decided to focus this work in the Ca^2+^-saturated forms of these proteins. Also, it is to be noted that apo-CaM has a much more reduced regulatory role in neuronal activity than Ca^2+^-saturated CaM, reviewed in [13]. The results of our previous experimental work of Aβ(1–42):CaM complexation led us to the conclusion that complexes with a 2:1 Aβ(1–42):calcium-binding protein complex stoichiometry are formed [16]. The results of this work also lend support to the formation of complexes with a 2:2 Aβ(1–42):calcium binding protein complex stoichiometry upon binding of a second molecule of CaM or calbindin-D28k to previously formed 2:1 Aβ(1–42):calcium binding protein complexes. Therefore, CaM and calbindin-D28k can buffer the free Aβ(1–42) concentration down to about 1 nM in neurons expressing both proteins. The kinetic analysis of the formation rate of these complexes points out that CaM binds Aβ(1–42) about two-fold more rapidly than calbindin-D28k. Thus, CaM will bind Aβ(1–42) more efficiently than calbindin-D28k. The fact that the rate of dissociation of Aβ(1–42) from Aβ(1–42):CaM complexes is approximately 10-fold lower than the rate of formation of this complex give strong support to the hypothesis that a conformational reorganization takes place after the initial formation of the complex. A direct inspection of the 24–42 amino acids domain of Aβ(1–42) in the model structures 1 and 2 (Figure 2B and Figure 3B) reveals its distortion with respect to the structure of this peptide domain in PDB ID: 1IYT and 1Z0Q files. These structures were obtained by Nuclear Magnetic Resonance spectroscopy in ‘helix-promoting’ environments [34,35], and the results of our previous work [16] pointed out that the binding pocket of Aβ(1–42) in CaM should be highly hydrophobic. As Aβ(1–42) equilibrium between α and β conformations represented by these PDB ID files is strongly dependent upon the polarity of the microenvironment [35], this structural distortion suggests that CaM binding shifts the equilibrium between α and β structures of Aβ(1–42). Indeed, the need for an early distortion of the 24–42 amino acids domain of Aβ(1–42) might also account for the lag phase of about 1 min observed in the formation of Aβ(1–42):calbindin-D28k complexes. Overall, our results suggest a significant conformational flexibility of the C-terminal domain of Aβ(1–42) upon binding to proteins. In addition, the docking analysis of the model structures of the Aβ(1–42):CaM complex point out that Lys28 or Lys16 of Aβ(1–42) are among the strongly interacting amino acid residues in the protein interface of the complex, it is likely that saline bridges further lock Aβ(1–42) in the complex. The ratio values between the half-times of formation and dissociation of Aβ(1–42):CaM and Aβ(1–42):calbindin-D28k complexes indicate that the dissociation constant of Aβ(1–42):calbindin-D28k complexes is around 10-fold higher than that of Aβ(1–42):CaM complexes. Due to this, we conclude that CaM will prevail over calbindin-D28k for the trapping of Aβ(1–42) concentrations in the nanomolar range in neurons expressing both proteins. 

The dissociation rate of Aβ(1–42) from Aβ(1–42):CaM and from Aβ(1–42):calbindin-D28k complexes is very slow in respect to the rate and frequency of many molecular events linked to repetitive synaptic activity. Since repetitive synaptic activity plays a key role in neuronal activity during learning and memory formation [36], and Aβ(1–42) oligomers have been shown to impair synaptic efficiency [1,13,37], complexation of Aβ(1–42) by CaM or calbindin-D28k can be seen as a protection mechanism against this stressor molecule in neurons. Interestingly, CaM-like skin protein, which is present at concentrations ranging between 3 and 6 nM in the human cerebrospinal fluid [38], affords efficient protection against spatial learning impairment in a mouse model of AD [39] and inhibits neuronal death in a cell-based AD model [40]. But CaM or calbindin-D28k complexation can also be seen as a mechanism for adaptive response to Aβ(1–42) oligomers-induced stress, because it will transduce the initial Aβ(1–42) oligomers stress into cell signaling mediated by Aβ(1–42):CaM, and also in neurons expressing calbindin-D28k, by Aβ(1–42):calbindin-D28k complexes. Furthermore, in a previous work we reported that CaM binds not only Aβ(1–42) oligomers but also other highly neurotoxic peptides derived from Aβ(1–42), like Aβ(25–35) [16]. Furthermore, Aβ(25–35) is the shortest that retains the toxicity of the full length peptide [41], and it has been suggested that this peptide is the biologically active region of Aβ [42]. Other authors have also pointed out that the Aβ(25–35) fragment is strongly neurotoxic, see for example [43,44,45,46]. In addition, using surface plasmon resonance Guo et al. [47] have shown that tau, another hallmark of AD, binds with nanomolar affinity to multiple Aβ peptides in the mid to C-terminal regions of Aβ. On these grounds, we decided to select the docking model of the Aβ(1–42):CaM complex displaying more interactions with the C-terminus domain of Aβ(1–42), as the template for the design of a peptide with a high shielding efficiency for the amyloid peptide. Next, we experimentally assessed its ability to antagonize the formation of the Aβ(1–42):CaM complex. 

**Figure 8:**
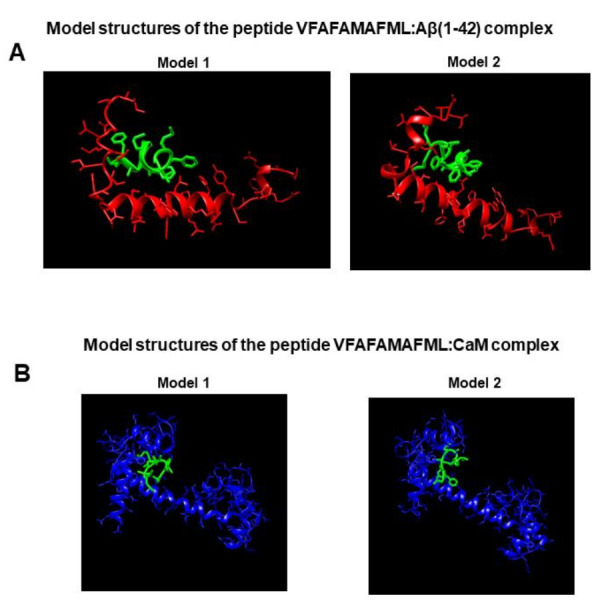
Highest-rank model structures of the complexes between the VFAFAMAFML peptide and Aβ(1–42) and CaM saturated with Ca^2+^ generated in silico by docking using the CABS-dock web server. (**A**) Model structures of the peptide VFAFAMAFML:Aβ(1–42) complex. (**B**) Model structures of the peptide VFAFAMAFML:CaM complex. The two model structures with higher cluster density are shown for each complex, i.e., the two most probable structures yielded by the CABS-dock web server. Peptide backbones and side chains of amino acids are shown. The VFAFAMAFML peptide is colored in green, Aβ(1–42) in red, and CaM in blue. See the Materials and methods section for more details.

The amino acid residues of CaM that docking simulations predicted to be directly involved in the interactions with the C-terminus domain of Aβ(1–42) are highly hydrophobic. Considering their spatial organization in the CaM structure, we included a couple of additional hydrophobic amino acid residues as spacing groups. The designed peptide (from N-terminus to C-terminus): VFAFAMAFML (amidated-C-terminus amino acid) demonstrated to be a potent inhibitor of the formation of both Aβ(1–42):CaM and Aβ(1–42):calbindin-D28k complexes. Thus, this result provides strong experimental support for the hypothesis that the initial formation of the Aβ(1–42):CaM complex is driven by hydrophobic interactions. To further strengthen this point, docking simulations of interactions of the VFAFAMAFML peptide with Aβ(1–42) and with the CaM conformation saturated by Ca^2+^ were performed using the CABS-dock web server as indicated in the Materials and methods section, without the need for any ‘a priori’ assumption of the peptide three-dimensional structure. The two most probable model structures yielded by this approach for the complexes between the VFAFAMAFML peptide and Aβ(1–42) and CaM are presented in the Figure 8. In addition, this in silico analysis yielded several other model structures closely related to these model structures 1 and 2 within the 10 most probable outcomes. Direct visual inspection of the model structures shown in the Figure 8 allows to realize that the VFAFAMAFML peptide is likely interacting with the interface domains of Aβ(1–42) and CaM that the ClusPro/PDBePISA approach predicted to be more strongly involved in the formation of Aβ(1–42):CaM complexes. This result provides a plausible hypothesis for the antagonism of the Aβ(1–42):CaM complexes formation by the VFAFAMAFML peptide. Also, this result suggests that the three-dimensional structure of the Aβ(1–42):CaM complex should be close to that used as a template model in this work. Because we measured in a previous study a dissociation constant of the Aβ(1–42):CaM complex near 1 nM [16], we focused on fluorescence, and we devised a method to monitor the formation of these complexes in the nanomolar range of concentrations. However, fluorescence spectroscopy cannot reach the atomic resolution level of protein complexes. On the other hand, experimental approaches which can be used for resolution of these complexes at an atomic level, like high-field Nuclear Magnetic Resonance or high-resolution Small-Angle X-Ray Scattering, requires the use of much higher concentrations of, both, Aβ(1–42) and CaM. Therefore, extensive experimental studies will be needed to resolve the three-dimensional structure of this complex at an atomic level, a point that is out of the scope of this work. 

However, other hydrophobic peptides with alternate sequences of amino acid cannot be excluded, due to the weak strength of this type of short-range interactions and, also, by their low selectivity to discriminate between different hydrophobic side chains of amino acid residues. This is illustrated by those amino acid residues of calbindin-D28k forming paired interactions with the 25–42 amino acid residues of Aβ(1–42) in the docking structures obtained for and Aβ(1–42):calbindin-D28k complexes. For example, the amino acid residues of calbindin-D28k with high BSA/ASA values in Aβ(1–42):calbindin-D28k complexes that are close to Ile31 of Aβ(1–42) varies in the three model structures yielded by docking simulations: Val173 in structure 1; Leu89 and Arg93 in structure 2; Asn157 and Gln182 in structure 3. Similarly, docking simulations give high BSA/ASA values in Aβ(1–42):calbindin-D28k complexes for the following amino acid residues of calbindin-D28k located in the proximity of Ile41 of Aβ(1–42): Ser28, Phe61 and Tyr30 in structure 1; Pro172 in structure 2; Ser28 and Phe177 in structure 3. This variability of calbindin-D28k amino acid residues with high BSA/ASA values in Aβ(1–42):calbindin-D28k complexes is also highlighted by the amino acid residues displaying spatial vicinity to the short side chain Gly37/38 amino acid residues of Aβ(1–42): Ser28 and Ser156 in structure 1; Pro172 and Leu89 in structure 2; Ser28, Asn157, Phe177, Ser156 and Gln182 in structure 3. Comparing the calbindin-D28k amino acid residues with those of CaM highlights the possibility of designing a family of alternate peptides that could antagonize the formation of Aβ(1–42):CaM and Aβ(1–42):calbindin-D28k complexes. However, it is to be noted that further experimental work is needed to validate the most probable structure(s) of the Aβ(1–42):calbindin-D28k complex. Indeed, two experimental results included in this work strongly argue against model structure 2 in Figure 7. First, the relatively high weight of the charged and polar amino acids residues of calbindin-D28k in the interface of model structure 2 is not consistent with the blockade of the formation of this complex by nanomolar concentrations of the strongly hydrophobic VFAFAMAFML (amidated-C-terminus amino acid) peptide. Second, the microenvironment of the HiLyteTM-Fluor555 dye attached to the N-terminus amino acid of Aβ(1–42) in this model structure 2 should not be significantly altered, which is against the conclusion derived from the fluorescence measurements. 

The VFAFAMAFML (amidated-C-terminus amino acid) peptide antagonist against the formation of Aβ(1–42):CaM and Aβ(1–42):calbindin-D28k complexes designed in this study is highly hydrophobic, and its small size allows it to be envisaged that it can be rapidly incorporated within the cells. Moreover, we have experimentally assessed that the incubation of HT-22 cells in culture for 24 h with 0.1–1 micromolar concentration of the antagonist peptide VFAFAMAFML (amidated-C-terminus amino acid) does not have any significant effect on cell viability (the data are included as supplementary material). Thus, it has a potential value for its therapeutic use by direct intracranial injection. However, the potential therapeutic use of this peptide by intravenous injections will require the evaluation of its permeability across the blood-brain barrier. In addition to the putative therapeutic use of the antagonist peptide developed and experimentally evaluated in this work, it may be useful for the evaluation of Aβ(1–42):CaM and Aβ(1–42):calbindin-D28k complexes relevance on the Aβ(1–42)-induced metabolic and functional dysregulation in brain neurons in culture. Moreover, this antagonist peptide provides the first template of amino acid residues sequence for the rational search for endogenous neuropeptides that can afford protection against Aβ(1–42)-induced neurotoxicity mediated by Aβ(1–42):CaM and Aβ(1–42):calbindin-D28k complexes. The role of some endogenous neuropeptides as neuroprotection agents against the development of AD is a promising line of research that has been highlighted in several publications, see e.g., [48,49]. Furthermore, data pointing out that ghrelin, neurotensin, pituitary adenylate cyclase-activating polypeptide (PACAP), neuropeptide Y, substance P, and orexin may be associated with the pathophysiology and potential therapy of Alzheimer’s disease has been reviewed in [49]. Interestingly, the amino acid residues of the N-terminus sequence of ghrelin are largely hydrophobic (UniProtKB—Q9UBU3 (GHRL_HUMAN)), a significant reduction of ghrelin mRNA has been reported in the temporal gyrus of AD patients [50] and it has been proved that a single polymorphism mutation leading to a Gln/Leu substitution is associated with the onset age of AD [51]. 

In summary, the major novel findings reported in this work are: (1) the complexation between the nanomolar concentrations of Aβ(1–42) and calbindin-D28k and (2) the submicromolar concentrations of the synthesized peptide VFAFAMAFML (amidated-C-terminus amino acid) designed in this work efficiently blocks the formation of Aβ(1–42):CaM and Aβ(1–42):calbindin-D28k complexes. The latter results point out that the highly hydrophobic peptide VFAFAMAFML (amidated-C-terminus amino acid) could be useful as a tool to antagonize Aβ(1–42):CaM and Aβ(1–42):calbindin-D28k complexes’ formation in brain cells.

## 4. Materials and Methods

### 4.1. Chemicals and Reagents

Human Aβ(1–42)-HiLyte™-Fluor555 was obtained from AnaSpec (Freemont, CA, USA). The unlabeled Aβ(1–42) were synthesized and supplied by StabVida (Caparica, Portugal) and GenicBio Limited (Shanghai, Popular Republic of China). The peptide VFAFAMAFML (amidated-C-terminus amino acid)—synthetized upon request and supplied by GenicBio Limited (Shanghai, Popular Republic of China)—was dissolved in dimethylsulfoxide (DMSO) at a concentration of 10 mM and stored at −20 °C until use. Purified bovine brain CaM, HisTrap^TM^ FF and a Thrombin Clean Cleavage^TM^ kit were purchased from Sigma-Aldrich (Madrid, Spain). Microcolumns NZYMiniPrep were acquired from NZYTech (Lisbon, Portugal).

All other reagents and chemicals were of analytical grade from Sigma-Aldrich (Madrid, Spain), Roche–Merck (Darmstadt, Germany), and ThermoFisher Scientific (Madrid, Spain).

### 4.2. Aβ(1–42) Solutions and Aggregation State

The Aβ(1–42) solutions were prepared by dissolving the solid lyophilized peptide in 1% NH_4_OH and diluted with phosphate-buffered saline to the desired concentration, as in [16]. The aggregation states of the Aβ(1–42) stock solutions used in this work were evaluated using the rapid photoinduced cross-linking of unmodified proteins approach as described in detail in [17]. Dimers are the predominant aggregation state of Aβ(1–42) in our 2 μM of stock solutions, with a minor (<10%) contribution of trimers and undetectable concentration of Aβ(1–42) monomers. 

### 4.3. Expression and Purification of Calbindin-D28k

The protocol for expressing and purifying calbindin-D28k was derived from [52] with minor modifications. We purchased an already cloned into pET15b codon-optimized cDNA for CALB1 expression in E. coli, with flanking restriction sites for NcoI and BamHI (GenScript, Piscataway, NJ, USA). The BL-21 (DE3) competent cells were transformed with 50 ng/mL of the recombinant plasmid pET-15b-CALB1 and transformants were platted in lysogeny broth (LB) agar supplemented with 100 μg/mL of ampicillin overnight at 37 °C. A single colony containing the recombinant plasmid was inoculated into 5 mL of LB media supplemented with ampicillin (100 µg/mL) and grown at 37 °C for 12 h under shaking. This culture was used to inoculate 1 L of the same media kept with the same growing conditions. Once the cellular optic density reached 0.6, protein expression was induced by adding 0.4 mM of isopropyl-β-D-thiogalactoside (IPTG) to the media, and the culture was kept under shaking at 200 rpm at 25 °C for 10–12 h.

Next, the cells were spined down by centrifugation at 6000× *g* for 30 min. The pellets were collected and resuspended in the following buffer: 150 mM of tris-(hydroxymethyl) aminomethane (Tris-HCl), 150 mM of NaCl, 50 mM of MgCl_2_, 1 mM of phenylmethylsulfonyl fluoride, and 1 mg/mL of lysozyme (pH 8), and kept under stirring for 90 min at 4 °C. The cells were lysed with two freeze/thaw cycles at −80 °C and at room temperature. After this point, 0.2% sodium deoxycholate was added to the media to ensure a better solubilization of the cellular content. The DNA released by the cells was removed by the addition of 0.05 mg/mL of DNAse. The lysate was centrifuged at 8000× *g* for 30 min, and the supernatant was collected and brought to pH 8.0. The recombinant CALB1 containing the poly His-tail protein was purified using affinity chromatography columns (HisTrap^TM^-FF). The supernatant was loaded onto a 10 mL prepacked HisTrap^TM^-FF chromatographical support equilibrated with a buffer of 20 mM of Tris-HCl, 0.5 M of NaCl, and 10 mM of imidazole (pH 7.4). After washing with a buffer (10 volumes), the proteins retained into the column were eluted with a buffer supplemented with 250 mM of imidazole. The almost pure recombinant calbindin-D28k was loaded onto a Sephadex G75 column (1 × 50 cm) equilibrated with 50 mM of Tris-HCl (pH 7.5) to further purify the protein from undesired contaminants. After this step, the protein purity was assessed by sodium dodecyl sulfate-polyacrylamide gel electrophoresis (SDS-PAGE) (Supplementary Figure S1) and the protein yield obtained after each one of the purification steps is listed in Table S1, included in Supplementary Figure S1. Purified protein batches were kept at −80 °C until use. 

The Poly-Histidine_6_ tag was cut by overnight incubation with a Thrombin Clean Cleavage^TM^ kit. The purified recombinant calbindin-D28k protein was separated from the generated peptide by chromatography using a Sephadex G75 column of 1 cm diameter × 25 cm length. The treatment efficiency for the removal of the poly-His_6_ tail was assessed by SDS-PAGE (Supplementary Figure S1). Calbindin-D28k concentration was determined using an extinction coefficient of 27,957–28,037 M^−1^cm^−1^ at 280 nm [53,54]. In this work, we have routinely obtained fractions of purified recombinant calbindin-D28k with concentrations in the range 15 and 20 μM per batch, i.e., between 0.48 and 0.64 mg of protein/mL.

### 4.4. Measurements of Aβ(1–42) HiLyte^TM^-Fluor555 Interaction with CaM and Calbindin-D28k

The fluorescence measurements were performed using a Fluoromax+ fluorescence Spectrophotometer (Jovin Yvon technologies) at room temperature (24–25 °C) in quartz cells of 1 cm light-pathlength, with excitation and emission slits set to 5 nm.

The measurements of the kinetics of interaction were performed by adding 10 nM of Aβ(1–42) HiLyte^TM^-Fluor555 in a buffer of 50 mM of *N*-[2-hydroxyethyl] piperazine-*N*′-[2-ethanesulfonic acid] (Hepes), 100 mM of KCl and 50 µM of CaCl_2_ (pH 7.05). The cuvette was kept with magnetic stirring in the dark within the cuvette holder of the fluorimeter until the stabilization of the fluorescence intensity, routinely between 20 and 40 min. Then, CaM or calbindin-D28k were added to the cuvette at the concentrations indicated in the figures, and the kinetics of the fluorescence intensity were recorded with excitation and emission wavelengths of 520 nm and 567 nm, respectively. 

### 4.5. In silico Docking Experiments

The docking experiments were performed using ClusPro, a web server that performs the docking of two proteins by sampling billions of conformations. This server performs the following computational steps: (i) rigid-body coupling by sampling trillions of conformations; (ii) the clustering base calculated the root mean square deviation (RMSD) of the 1000 structure with the lowest energy generated, to find the clusters that will represent the most probable models of the complex; (iii) the refinement of the selected structure using energy minimization. The following PDB files were used for the determination of the interacting interface between Aβ(1–42) and CaM and calbindin-D28k:CaM saturated with calcium (PDB ID:1CLL), calbindin-D28k saturated with calcium (PDB ID: 6FIE), and the Aβ(1–42) structures (PDB ID:1IYT and/or 1Z0Q as indicated for each case). Due to the reported conformational changes in the C-terminus domain of Aβ(1–42) induced by changes in the microenvironment’s hydrophobicity [35], we have used two PDB ID files for Aβ(1–42), i.e., PDB ID 1Z0Q and 1IYT, as representative of the Aβ(1–42) conformations predominant in water and in a strongly hydrophobic environment, respectively. The top 10 highest ranked models for complex formation were selected for initial analysis in order to quantify the amino acid residues present at the interface. Interface analysis was performed with PDBe PISA (Protein, Interface, Surface, and Assemblies), available at https://www.ebi.ac.uk/pdbe/pisa. Last access for interface analysis of the Aβ(1–42):CaM complex on 11 November 2019, and last access for interface analysis of Aβ(1–42):calbindin-D28k complex on 3 January 2022. The hidden or buried surface area/accessible surface area ratio (BSA/ASA ratio) of the most likely complex simulations were used to quantify the involvement degree of each amino acid residue in the formation of the complex. A threshold level of 0.5–0.6 was chosen to select the amino acid residues that strongly contributed to the interacting surface. Plots and molecular analysis were performed with the UCSF Chimera package.

The modeling of protein-peptide interactions was performed using the CABS-dock web server (http://biocomp.chem.uw.edu.pl/CABSdock, accessed on 1 February 2022) [55,56], as in a previous work [16]. The PDB files used in this docking analysis are: 1CLL for CaM saturated with Ca^2+^ and 1IYT for Aβ(1–42). 

### 4.6. Experimental Evaluation of the Efficiency of the Designed Peptide to Antagonize the Interaction between Aβ(1–42) HiLyte^TM^-Fluor555 and CaM or Calbindin-D28k

The measurements of the experimental evaluation of the efficiency of the designed peptide were carried out with 10 nM of Aβ(1–42) HiLyte^TM^-Fluor555 preincubated for 10–15 min with different concentrations of the peptide in buffer 50 mM of Hepes, 100 mM of KCl, and 50 μM of CaCl_2_ (pH 7.05) before the addition of CaM or calbindin-D28k. The peptide VFAFAMAFML (amidated-C-terminus amino acid) was added from concentrated stock solutions in DMSO, taking care that the DMSO concentration was always between 0.05 and 0.1% in the cuvette of fluorescence measurements. The kinetic assays were made with excitation and emission wavelengths of 520 nm and 567 nm, and the kinetics of fluorescence were recorded after the addition of CaM or calbindin-D28k at the concentrations indicated in the figures’ captions. Experiments run with 0.05 and 0.1% DMSO showed no effect of this DMSO concentrations on these kinetics of fluorescence in the absence of the peptide.

### 4.7. Statistical Analysis

The results were expressed as the mean standard error (S.E.). Statistical analysis was carried out by the Student t-test. A significant difference was accepted at the *p* < 0.05 level. All results were confirmed in triplicate experiments.

## Figures and Tables

**Table 1:** A list of the strongly interacting amino acid residues of Aβ(1–42) and CaM in structures 1 and 2 yielded by docking simulations for the 1:1 Aβ(1–42):CaM complex. The amino acid residues of both Aβ(1–42) and CaM are listed from high to low values of the parameter BSA/ASA (values in parenthesis), obtained from the interface analysis with PDBePISA software. Only amino acids with BSA/ASA values ≥0.5 are listed.

	Aβ(1–42) Amino Acid Residues	CaM Amino Acid Residues
Model structure 1	Gly37 (1.0), Gly38 (1.0), Val36 (0.98), Phe20 (0.97), Val40 (0.89), Leu34 (0.83), Val39 (0.82), Met35 (0.77), Lys28 (0.72), Val24 (0.66)	Ile27 (1.0), Leu32 (1.0), Ile52 (1.0), Ile63 (1.0), Phe68 (1.0), Met71 (1.0), Phe19 (0.99), Phe89 (0.99), Gly98 (0.98), Val55 (0.89), Met51 (0.88), Arg86 (0.85), Met36 (0.76), Met72 (0.74), Lys75 (0.73), Thr79 (0.70), Gly96 (0.68), Asn97 (0.64)
Model structure 2	Val39 (0.99), Ala42 (0.99), Gly38 (0.98), Met35 (0.96), Ile41 (0.82), Val24 (0.79), Lys16 (0.5)	Val136 (1.0), Phe141 (1.0), Phe92 (0.997), Leu105 (0.99), Ala88 (0.90), Glu87 (0.85), Leu112 (0.77), Met124 (0.63), Met109 (0.61), Val91 (0.57), Met145 (0.56), Ala128 (0.52)

**Table 2:** List of strongly interacting amino acid residues of Aβ(1–42) and calbindin-D28k in the selected model structures 1, 2 and 3 yielded by docking simulations for the 1:1 Aβ(1–42):calbindin-D28k complex. The amino acid residues of both Aβ(1–42) and calbindin-D28k are listed from high to lower values of the parameter BSA/ASA (values in parenthesis) obtained from the interface analysis with PDBePISA software. Only amino acid residues with BSA/ASA values ≥0.5 for Aβ(1–42) and ≥0.6 for calbindin-D28k are listed.

	Aβ(1–42) Amino Acid Residues	Calbindin-D28k Amino Acid Residues
Model structure 1	Val24 (1), Phe20 (0.98), Leu34 (0.94), Asp23 (0.93), Leu17 (0.91), His14 (0.90), Val18 (0.88), Gly38 (0.86), Ile31 (0.84), Ala21 (0.65), Ile41 (0.63), Gly33 (0.62), Ala30 (0.55), Gly37 (0.52)	Val173 (0.96), Ile73 (0.94), Val181 (0.88), Leu179 (0.86), Lys72 (0.85), Glu177 (0.85), His80 (0.84), Ser28 (0.83), Glu57 (0.82), Phe61 (0.81), Pro172 (0.68), Tyr30 (0.67), Ser156 (0.61)
Model structure 2	Gly33 (0.98), Gly37 (0.96), Ile31 (0.91), Phe20 (0.86), Asp23 (0.78), Leu17 (0.75), Ala30 (0.75), Leu34 (0.74), His13 (0.68), Lys16 (0.66), Ala42 (0.63), Ile41 (0.55), Val36 (0.54)	His80 (0.95), Arg47 (0.95), Pro172 (0.92), Leu89 (0.82), Glu57 (0.78), Arg93 (0.77), Leu82 (0.76), Lys98 (0.74), Ser55 (0.74), Glu77 (0.73), Pro83 (0.72), Leu52 (0.71), Val81 (0.63)
Model structure 3	Gly37 (0.99), Ala21 (0.95), Ile31 (0.92), Leu17 (0.80), Ile41 (0.78), Leu34 (0.76), Gly38 (0.71), His14 (0.69), Val40 (0.63), Phe20 (0.62), Ala30 (0.53), Ala42 (0.52),	Gly188 (0.99), Ser28 (0.95), Asn157 (0.87), Ala258 (0.84), Glu166 (0.83), Phe177 (0.80), Asn192 (0.72), Cys257 (0.70), Arg249 (0.70), Ser156 (0.67), Phe191 (0.67), Thr250 (0.63), Gln182 (0.63)

## Data Availability

Not applicable.

## Supplementary Materials

The following are available online at https://www.mdpi.com/article/10.3390/ijms23042289/s1.

## Abbreviations

Aβ	amyloid β
AD	Alzheimer’s disease
BSA/ASA	buried surface area/accessible surface area ratio
CaM	calmodulin
DMSO	dimethylsulfoxide
Hepes	*N*-[2-hydroxyethyl] piperazine-*N*′-[2-ethanesulfonic acid]
IC50	concentration producing 50% of the maximum effect
IPTG	isopropyl-β-d-thiogalactoside
LB	lysogeny broth
Qmax	maximum extinction of fluorescence
SDS-PAGE	sodium dodecyl sulfate-polyacrylamide gel electrophoresis
S.E.	standard error
Tris	tris-(hydroxymethyl) aminomethane
