# Supplementary material for: Design and Experimental Evaluation of a Peptide Antagonist against Amyloid β(1–42) Interactions with Calmodulin and Calbindin-D28k
Source: Int J Mol Sci. 2022 Feb 18;23(4):2289. doi: 10.3390/ijms23042289 (PMC8880779; doi:10.3390/ijms23042289)
Supplement: Supplementary file 1 [file ijms-23-02289-s001.zip › ijms-1584004-supplementary.pdf]

Supplementary Table S1. Recombinant calbindin-D28k purification. Recombinant calbindin-D28k purification. Data showing the yield obtained after each one of the purification steps in one of the three purifications batches of recombinant calbindin-D28k performed in this work.

Step	Protein (mg/mL)	Volume (mL)	Protein (mg)	Yield (%)
Cell lysate	99.8	17.5	1747	100
Supernatant	35.2	21.3	750	43.0
His Trap™ FF	0.96	50	48	2.7
Sephadex G75	0.70	60	42	2.4

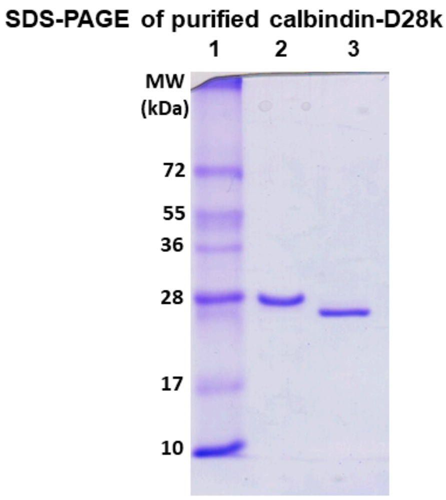

Supplementary Figure S1. SDS-PAGE of purified calbindin-D28k. SDS-PAGE 12.5% acrylamide gel of purified calbindin-D28k. Lane 1: molecular weight (MW) markers. Lane 2: 0.35 µg of purified calbindin-D28k collected after the final Sephadex G75 step. Lane 3: 0.3 µg of purified calbindin-D28k after removal of the polyHis6 tail by treatment with the Thrombin Clean CleavageTM kit as indicated in the Materials and Methods.

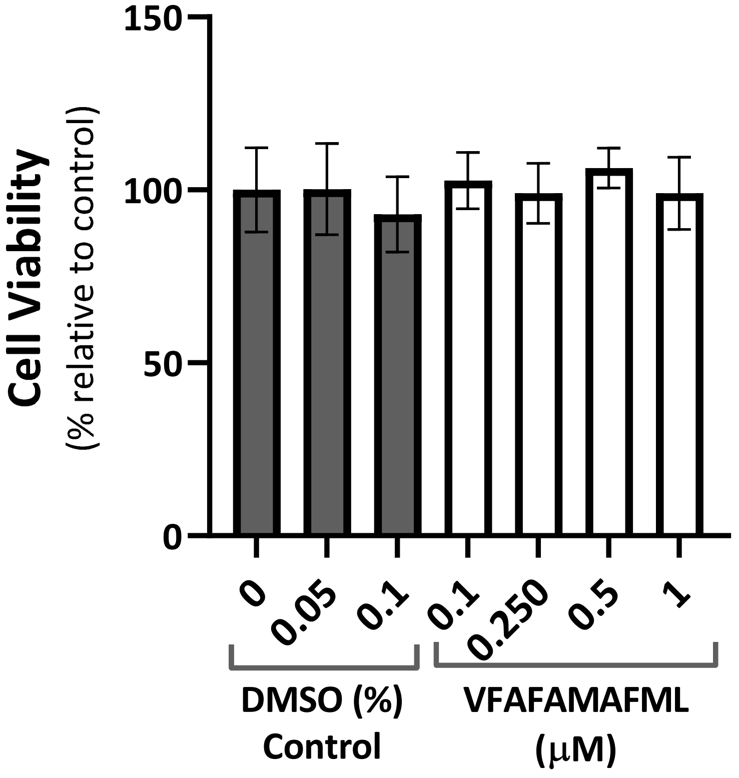

Supplementary Figure S2. The cell viability of the mouse hippocampal HT-22 cell line was not significantly affected by the VFAFAMAFML peptide at the concentrations tested (0.1-1 µm) after 24h incubation. Cell viability assay was measured as described in previous works [17], see also [Marques-da-Silva, D. and Gutierrez-Merino, C. Caveolin-rich lipid rafts of the plasma membrane of mature cerebellar granule neurons are microcompartments for calcium/reactive oxygen and nitrogen species cross-talk signaling. Cell Calcium (2014) 56, 108–123; Fortalezas S., Marques-da-Silva, D. and Gutierrez-Merino, C. Creatine Protects Against Cytosolic Calcium Dysregulation, Mitochondrial Depolarization and Increase of Reactive Oxygen Species Production in Rotenone-Induced Cell Death of Cerebellar Granule Neurons. Neurotox. Res. (2018) 34, 717–732]. Briefly, HT-22 were seeded in 96-well plates at a density of 8.8 x103 cells/cm2 in the culture media (Dulbecco's modified Eagle's medium (DMEM)-high glucose, supplemented with 10 % inactivated fetal bovine serum (FBS), 4 mM glutamine, 1 mM pyruvic acid, 100 U/mL penicillin and 100 µg/mL streptomycin) and allowed to grow at 37°C and 5% CO2. After 48 h of cell growth, different concentrations of the peptide VFAFAMAFML (0.1-1 µM) were incubated in the culture media for 24 h at 37°C and 5% CO2. Vehicle controls were performed with 0.05 and 0.1 % of DMSO, the maximum concentrations of DMSO used in the cell viability assay. Then cells were washed with 1 mL of buffered medium (pH 7.4) at 37ºC and incubated with 0.3 mg/ml of 3 - (4,5 - dimethylthiazol - 2 - yl) - 2,5 - diphenyltetrazolium bromide (MTT) solution for 20 min at 37º C and 5% CO2. Next, the supernatant was discarded and 100 µL of DMSO was added to solubilize the purple formazan product produced by cells. The amount of colored formazan formed by the reduction of MTT was measured spectrophotometrically at 490 nm (wavelength of reference = 700nm). Untreated cells (0% DMSO) were regarded as controls (100% cell survival) and the cell survival ratio was expressed as the percentage of the control. The results were the average ± s.e. of experiments done in triplicate in three independent assays.
